# A novel role for ezrin in breast cancer angio/lymphangiogenesis

**DOI:** 10.1186/s13058-014-0438-2

**Published:** 2014-09-18

**Authors:** Abdi Ghaffari, Victoria Hoskin, Alvin Szeto, Maaike Hum, Navid Liaghati, Kanji Nakatsu, Yolanda Madarnas, Sandip Sengupta, Bruce E Elliott

**Affiliations:** 1 Department of Pathology and Molecular Medicine, Queen's University, Rm 324 18 Stuart Street, Kingston, K7L 3N6 ON Canada; 2 Division of Pharmacology and Toxicology, Department of Biomedical and Molecular Sciences, Queen's University, 18 Stuart Street, Kingston, K7L 3N6 ON Canada; 3 Department of Laboratory Medicine, The Credit Valley Hospital & Trillium Health Centre, 2200 Eglinton Avenue West, Mississauga, L5M 2N1 ON Canada; 4 Department of Oncology, Queen's University, 10 Stuart Street, Kingston, K7L 3N6 ON Canada; 5 Division of Cancer Biology and Genetics, Cancer Research Institute, Queen's University, Rm 302C, 10 Stuart St., Kingston, K7L 3N6 ON Canada

## Abstract

**Introduction:**

Recent evidence suggests that tumour lymphangiogenesis promotes lymph node metastasis, a major prognostic factor for survival of breast cancer patients. However, signaling mechanisms involved in tumour-induced lymphangiogenesis remain poorly understood. The expression of ezrin, a membrane cytoskeletal crosslinker and Src substrate, correlates with poor outcome in a diversity of cancers including breast. Furthermore, ezrin is essential in experimental invasion and metastasis models of breast cancer. Ezrin acts cooperatively with Src in the regulation of the Src-induced malignant phenotype and metastasis. However, it remains unclear if ezrin plays a role in Src-induced tumour angio/lymphangiogenesis.

**Methods:**

The effects of ezrin knockdown and mutation on angio/lymphangiogenic potential of human MDA-MB-231 and mouse AC2M2 mammary carcinoma cell lines were examined in the presence of constitutively active or wild-type (WT) Src. *In vitro* assays using primary human lymphatic endothelial cells (hLEC), an *ex vivo* aortic ring assay, and *in vivo* tumour engraftment were utilized to assess angio/lymphangiogenic activity of cancer cells.

**Results:**

Ezrin-deficient cells expressing activated Src displayed significant reduction in endothelial cell branching in the aortic ring assay in addition to reduced hLEC migration, tube formation, and permeability compared to the controls. Intravital imaging and microvessel density (MVD) analysis of tumour xenografts revealed significant reductions in tumour-induced angio/lymphangiogenesis in ezrin-deficient cells when compared to the WT or activated Src-expressing cells. Moreover, syngeneic tumours derived from ezrin-deficient or Y477F ezrin-expressing (non-phosphorylatable by Src) AC2M2 cells further confirmed the xenograft results. Immunoblotting analysis provided a link between ezrin expression and a key angio/lymphangiogenesis signaling pathway by revealing that ezrin regulates Stat3 activation, VEGF-A/-C and IL-6 expression in breast cancer cell lines. Furthermore, high expression of ezrin in human breast tumours significantly correlated with elevated Src expression and the presence of lymphovascular invasion.

**Conclusions:**

The results describe a novel function for ezrin in the regulation of tumour-induced angio/lymphangiogenesis promoted by Src in breast cancer. The combination of Src/ezrin might prove to be a beneficial prognostic/predictive biomarker for early-stage metastatic breast cancer.

**Electronic supplementary material:**

The online version of this article (doi:10.1186/s13058-014-0438-2) contains supplementary material, which is available to authorized users.

## Introduction

The overexpression and abnormal localization of ezrin, the founding member of the ezrin-radixin-moesin (ERM) family of membrane cytoskeletal crosslinkers [1], has been associated with positive LN status, metastasis, and poor outcome in various human cancers including breast [2]-[5]. Growing evidence suggests ezrin and moesin as novel prognostic markers of disease outcome [6],[7], although the molecular and cellular basis of their role in breast cancer remains unclear. ERMs are expressed in a tissue-specific manner, with ezrin predominately expressed in epithelial cells, suggesting different ERM functions in specific cell types [1]. Ezrin interacts with several cell signaling molecules involved in tumour progression including hepatocyte growth factor (HGF) receptor Met, β4-integrin, and Src family kinases [8]. Ezrin is the only ERM protein to be directly phosphorylated by Src kinase at tyrosine 477, which has been shown to induce a phospho-specific association between ezrin and its binding partners [9],[10]. In addition, ezrin Y477 phosphorylation is required for the Src-induced invasive phenotype of cells in three-dimensional matrix [11]. We have previously shown that the expression of the ezrin Y477F mutant, non-phosphorylatable by Src, significantly reduced spontaneous lung metastasis in a mammary fat pad engraftement model [12].

Src is a non-receptor tyrosine kinase that is commonly deregulated in many human cancers and plays a crucial role in tumorigenesis and metastasis [13]. Src is commonly hyper-activated in human cancers and promotes metastasis in part by inducing tumour angiogenesis via a signal transducer and activator of transcription 3 (Stat3)/vascular endothelial growth factor (VEGF)-A signaling pathway [13]. However, the role of Src in the regulation of VEGF-C, tumour-induced lymphangiogenesis, and lymphovascular invasion (LVI) remains unclear. As ezrin is a key regulator of Src activity [14]-[16], we examined the potential role of Src/ezrin in tumour-induced angio/lymphangiogenesis in breast cancer. To address this notion, we initially assessed the effect of ezrin knockdown (KD) on angio/lymphangiogenic potential of human MDA-MB-231 (MDA231) cells expressing activated Src. Ezrin-deficient MDA231 cells demonstrated significant reduction in Src-induced neovascularization. Furthermore, the expression of ezrin Y477F reduced the angio/lymphangiogenic potential of the highly invasive mouse AC2M2 mammary carcinoma cell line. Our results suggest that ezrin promotes angio/lymphangiogenic activity by regulating Stat3 activation and expression of VEGF-A/-C and interleukin-6 (IL-6). These findings implicate a novel regulatory role for ezrin in Src-induced tumour vascularization and provide a mechanistic link between Src/ezrin expression and increased LVI and metastasis in breast cancer.

## Methods

### Cell lines

Primary human lymphatic endothelial cells (hLEC) were purchased from Lonza (CC-2812, Walkersville, MD, USA) and maintained in endothelial cell growth medium provided by the supplier (EGM-2 MV, CC-3156) or basic endothelial cell growth media (MCDB 131, Sigma-Aldrich, St. Louis, MO, USA) at 5% CO_2_ and 37°C and used at passages 2-5. The human basal-like breast cancer cell line MDA-MB-231 (MDA231) was a gift from Dr. Peter Siegel (McMaster University) and maintained in Dulbecco's modified Eagle's medium (DMEM, Invitrogen, Burlington, ON, Canada) with 10% fetal bovine serum (FBS) at 5% CO2 and 37°C. The MDA231 cell line expressing pWZL-hygro (empty vector) or constitutively active Y527F Src (MDASrc) were a gift from Dr. Alan Mak (Queen's University) [17] and maintained in DMEM with 10% FBS and selected with 100 μg/ml hygromycin. The AC2M2 cell line is a highly metastatic lung tumour variant selected from a CBA/J mouse-derived breast carcinoma cell line (SP1) following three serial intra-mammary injections of a lung metastatic nodule as described previously (29). Two independent lentiviral ezrin short hairpin RNA (shRNA) constructs expressed by a pLKO.1 vector (Target sequence for shEZR-1: CCTGGAAATGTATGGAATCAA; and shEZR-2: CCCACGTCTGAGAATCAACAA) were purchased from Open Biosystems (Thermo Fisher Scientific, Waltham, MA, USA) and used according to the manufacturer's instructions. Transduced cells were selected by the addition of 4 μg/ml puromycin to the growth medium. Both constructs demonstrated significant KD, however the shEZR-1 construct consistently achieved higher ezrin KD (>90%) and was used for the majority of experiments. Ectopic expression of the mutant Y477F ezrin and empty pCB6 vector in AC2M2 cells is previously described (23). Both pCB6 and Y477F ezrin-expressing AC2M2 cells were maintained in DMEM with 10% FBS plus 400 μg/ml G418 (Invitrogen) to select stable transfectants. Transient transfection of pCB6 empty vector and wild-type (WT) ezrin (EZR/WT) were performed using FugeneHD reagent (Promega, Madison, WI, USA) according to the manufacturer's protocol. Furthermore, AC2M2 cells were transfected with 10 nM non-silencing control or ezrin small interfering RNA (siRNA) using Lipofectamine 2000 reagent and Opti-MEM-I reduced serum medium (Invitrogen) as instructed by the manufacturer. A pool of three siRNAs against mouse ezrin (ON-TARGETplus SMARTpool, mouse VIL2, L-046568-01-0005) and non-targeting siRNA (ON-TARGETplus non-targeting siRNA #2, D-001810-02-05) were purchased from Thermo Fisher Scientific. siRNA KD of ezrin was confirmed by western blot. To collect conditioned medium (CM), cells were grown to 90% confluency in 100 mm culture dishes in DMEM plus serum. After washing with sterile serum-free media, cells were incubated in serum-free DMEM at 37°C for 24 hours. The CM was then collected, centrifuged at 3,000 g for 10 minutes, and concentrated through a 5 kiloDalton (kDa) filter.

### Cell proliferation assay

Cell growth *in vitro* was assessed using the 3-(4,5-dimethylthiazol-2-yl)-2,5-diphenyl tetrazolium bromide) (MTT) assay. Following seeding of equal number of cells, MTT assay was performed according to manufacturer's specifications (Sigma-Aldrich, Oakville, ON, Canada) at 24, 48, and 72 hours. Values represent mean O.D. (570 nm) ratios of eight wells relative to day 1.

### Western blotting

Western blot analysis was performed as described previously (23). Blots were blocked with 5% skim milk or bovine serum albumin (BSA) and probed with anti-VEGF-A and -C (R&D Systems, Minneapolis, MN, USA), anti-ezrin and anti-phospho-Thr ezrin (pT567)/Radixin (pT564)/Moesin(pT558) (pTERM, Cell Signaling, Beverly, MA, USA), anti-Src and anti-pY416 Src (Cell Signaling), anti-Stat3 and anti-pY705 Stat3 (Cell Signaling), anti-IL-6 (PeproTech, Rocky Hill, NJ, USA) and anti-γ-tubulin (Sigma-Aldrich) antibodies and the appropriate secondary antibodies. For analysis of Src activity, we used soluble/insoluble cell lysate fractions as described previously [16]. In brief, the soluble fraction was first extracted by a 1-min incubation with csk buffer (50 mmol/l MES, 3 mmol/l EGTA, 5 mmol/l MgCL_2_, 0.5% Triton X-100, pH 604). The remaining cellular material (insoluble fraction) was rinsed quickly with cold csk buffer, and extracted with 2 × laemmli buffer. Recombinant human IL-6 was obtained from R&D Systems. ImageJ software was used for densitometry analysis to calculate ratio of target bands to loading control (γ-tubulin).

### hLEC migration, tube formation, and permeability assays

*Migration assay*: Primary hLEC (2 × 10^4^) were seeded onto collagen-coated Transwell permeable inserts (8-μm pore size, Corning, Tewksbury, MA, USA) and co-cultured with 1 × 10^5^ MDASrc or MDASrc shEZR cells seeded on the companion plate. The co-culture system was incubated in 50:50 serum-free MCDB-131:DMEM media at 37°C for six hours, at which time the hLEC cells on top of the insert were removed by cotton swabs and the membrane was cut, fixed, and stained in DAPI. Multiple fields from each membrane were imaged using a fluorescent microscope (Olympus BX51, Olympus, Tokyo, Japan) and the number of nuclei counted using ImageJ software.

*Tube Formation Assay*: hLEC were seeded at 1 × 10^5^ cells in μ-Slide Angiogenesis plates (ibidi, München, Germany) in 50 μl EGM-2MV media atop of 10 μl of growth factor-reduced (GFR) Matrigel (BD Biosciences, Burlington, ON, USA) and were stimulated with CM from various tumour cell lines for four to six hours. Tube formations was imaged by a phase-contrast microscope (Olympus CKX41) and manually counted (n = 3).

*Permeability Assay*: Diffusion of TMR-dextran (average mol. wt. 2000 kDa, Invitrogen) through the intercellular spaces of the lymphatic endothelial junctions was used as a measure of macromolecular permeability across the lymphatic barrier [18]. To form a lymphatic barrier, hLEC (5 × 10^4^) were seeded onto collagen-coated Transwell inserts (8-μm pore size) 48 hours prior to overnight co-culture with 1 × 10^5^ MDASrc or MDASrc shEZR cells seeded on the companion 24-well plate. The insert was then removed and placed inside a new 24-well plate with fresh media and the permeability of the hLEC layer was measured using a fluorescence reader (Molecular Devices, Spectra Gemini XS, Sunnyvale, CA, USA) based on the amount of TMR-dextran that diffused through the lymphatic barrier into the lower compartment in 60 and 90 minutes.

### Ex vivo *aortic ring angiogenesis assay*

The aorta was harvested from Sprague-Dawley rats as described previously [19], cut into 0.5 mm ring segments and embedded in GFR-Matrigel in a 24-well cell culture plate. Embedded rings were covered in F12K media containing, 1% fetal bovine serum (FBS), 1% penicillin/streptomycin cocktail, and stimulated with CM from the various tumour cell lines. Photographs were taken every 24 hours using a phase-contrast microscope (Olympus CKX41) for up to 96 hours. Selected rings were stained by immunofluorescence (IF) for endothelial cell marker CD31 (Santa Cruz Technology, Santa Cruz, CA, USA, #sc-1506). Endothelial cell sprouting was quantified using ImagePro software.

### In vivo *Matrigel plug assay*

*In vivo* analysis of angio/lymphangiogenesis was performed by the Matrigel plug assay as previously described [20]. All animal procedures were carried out according to the guidelines of the Canadian Council on Animal Care with the approval of the Queen's University Animal Care Committee. A total of 3 × 10^4^ MDASrc or MDASrc shEZR were mixed with 200 μl of cold GFR-Matrigel and injected subcutaneously (s.c.) into six- to eight-week-old Rag2^-/-^II2rγ ^-/-^ double-knockout alymphoid (Rag2γ) mice, kindly provided by Dr. C. Tayade, (originally developed by Dr. M. Ito at the Central Institute for Experimental Animals, Kawasaki, Japan) or nude (NCr-Foxn1^nu/nu^) mice bred in house (Queen's University Animal Care Services). Each mouse acted as its own control (injecting left and right flanks) to minimize inter-subject variability. After 12 days, the intravital imaging of blood vasculature within the Matrigel plugs was performed using a Quorum WaveFX-X1 spinning disk confocal system (Quorum Technologies Inc, Guelph, ON, Canada). Mice were anesthetized with ketamine (150 mg/kg) and xylazine (10 mg/kg) and the jugular vein was cannulated for injection of FITC-albumin (Sigma-Aldrich) dye to visualize vasculature. Matrigel plugs were exposed using skin flap surgery and placed on the microscope stage for imaging. Following intravital imaging, Matrigel plugs were harvested and digitally photographed with a minimum of five plugs per group. Plugs were then fixed in 4% paraformaldehyde and paraffin-embedded for future sectioning. Syngeneic engraftment of AC2M2 cells (WT, Y477F, and siRNA KD) were performed in CBA/J mice as described above with 1 × 10^4^ cells per injection site. Matrigel plugs containing ezrin siRNA KD cells were harvested for analysis on day 10 post transfection. In all Matrigel plug assays, microvascular density (MVD) for each plug was assessed by manual counting of hematoxylin and eosin (H&E)-stained sections with at least three independent 'hot spots' (areas with the highest number of microvessel profiles at × 200 magnification) by a trained pathologist (N.L.) blinded to the study. In addition, the number of CD31-positive vessels within each Matrigel plug was counted from at least three independent hot spots (×400 magnification) to confirm histology scores. To obtain an average vascular size, the relative cross-sectional area of microvessels from at least three independent hot spots per plug (×400 magnification) were tracked in ImageJ software and reported in pixel^2^. To assess *in vivo* lymphangiogenesis, the relative fluorescent intensity of Lyve-1- and podoplanin-positive cells within at least three independent fields in each plug were calculated using ImageJ software.

### Tissue microarray analysis

With the Queen's University Research Ethics Board approval, breast tumour specimens were collected from 63 consenting female patients who received treatment for breast cancer at the Cancer Centre of Southeastern Ontario at Kingston General Hospital between 2005 and 2007. Clinicopathological data (for example LVI status) for each patient was retrospectively obtained from electronic and paper medical files by a clinical oncologist. The cohort was limited to premenopausal women that were 49 years of age or younger at the time of diagnosis, had primary invasive mammary carcinomas (infiltrating ductal and/or lobular), and staged within T1-3, N0-1, and M0, indicators of tumour size, nodal status, and metastases, respectively. Patients with any previous history of cancer, bilateral breast disease or neoadjuvant chemotherapy were excluded. Archival reduction mammoplasties from 20 consenting individuals were included as non-malignant controls.

### Immunohistochemistry and immunofluorescence

Immunohistochemical (IHC) and IF staining were performed as described previously [12]. The following antibodies were used overnight at 4°C: zonula occludens-1 (ZO-1) (Invitrogen, #901200), pan ezrin (Cell Signaling, #3145), pan Src (Cell Signaling, #2109), CD31 (Santa Cruz, #sc-1506), Lyve-1 (Merck Millipore, Darmstadt, Germany #ab2988), and AE1/AE3 cytokeratin (Santa Cruz, #sc-81714). Rabbit anti-mouse podoplanin antibody was a gift from Dr. Dontscho Kerjaschki (Medical University of Vienna, Austria) [21]. For mouse whole tissue section, images were obtained using a Quorum Wave FX spinning disk confocal microscope (Quorum Information Technologies Inc, Calgary, AB, Canada). Staining of the tissue microarray (TMA) was performed using an automated processor (Ventana Discovery XT; Ventana Molecular Discovery Systems, Tucson, AZ, USA) and automated quantification analysis (AQUA) was carried out as previously described [22]. IHC and IF images were acquired from the stained TMA sections by scanning with a ScanScope FL instrument, respectively (Aperio Technologies, Vista, CA, USA). AQUA scores for each target were obtained as described previously [23]. In brief, IF images were used to create 'masks' for cytokeratin and DAPI signals to assess the targets (Src, Ezrin) in epithelial tumour areas annotated by a pathologist. The signal intensity of the target was then quantified within each compartment to generate an AQUA score, which represents the sum of the pixel intensities for of the Src or Ezrin, divided by the area of the mask and normalized for exposure time. For IF staining of TMA sections, α-rabbit EnVision + HRP-labeled polymer (Dako, Burlington, ON, USA) and Cy5 Tyramide (PerkinElmer, Waltham, MA, USA) were used to amplify the target signals.

### Statistical analysis

Data were expressed as mean ± standard deviation (SD) and analyzed with an unpaired two-tailed *t* test (between two groups) or one-way analysis of variance with a Tukey-Kramer multiple comparison post test. In analyzing TMA results, both continuous and dichotomized (median used as cutoff point for high and low expression) AQUA scores were used. Unpaired *t* tests were used to assess associations between continuous AQUA scores and dichotomized clinical factors (for example LVI). *P* values less than 0.05 were considered statistically significant.

## Results

### Ezrin KD inhibits lymphangiogenic activity of breast carcinoma cells

**Figure 1:**
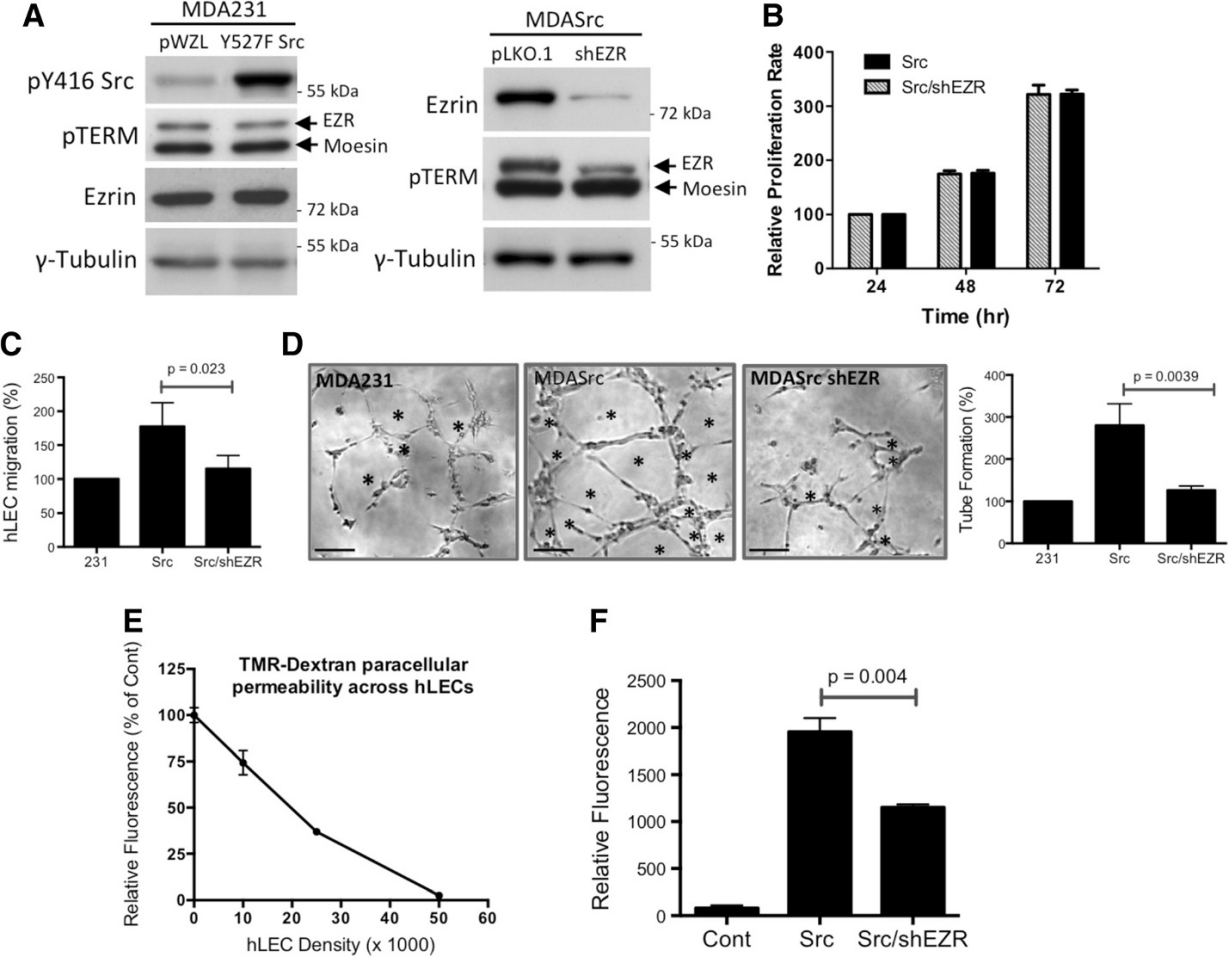
**Ezrin KD inhibits lymphangiogenic activity of breast cancer cells** ***.*** **(A)** The expression levels of active Src (pY416), pT567 ezrin/pT558 moesin (pTERM) and total ezrin in MDA231, MDASrc (Y527F Src in pWZL vector) and MDASrc shEZR (shRNA in pLKO.1 vector) cells were validated by western blot. Over 85% of endogenous ezrin was depleted in our shEZR cells. **(B)** The relative rates of proliferation (normalized against 24 hr) of MDA231 (231), MDASrc (Src) and MDASrc shEZR (Src/shEZR) cells were assessed by an MTT assay. **(C)** hLEC migration assay: percent migration of hLEC, co-cultured with MDA231 (231), MDASrc (Src), or MDASrc shEZR (Src/shEZR) cells (6 hr), is presented following normalization against the MDA231-treated group. **(D)** Tube formation assay: hLEC were stimulated with CM from MDA231, MDASrc, and MDASrc shEZR for 4 hr prior to manual counting of the tube-like structures (marked by asterisks). The histogram displays the percentage of tube formation in each group relative to MDA231 control cells. Scale bar = 50 μm. **(E)** The integrity of the hLEC monolayer was assessed by the paracellular diffusion of TMR-dextran (2000 kDa). Readings are expressed in relative fluorescence as percentage of control (collagen-coated membrane alone) (n = 3). **(F)** Monolayer of hLECs (5 × 10^5^ cells) was co-cultured overnight with no cells (control), MDASrc, or MDASrc shEZR (shEZR). The diffusion of TMR-dextran across the hLEC barrier was measured in 90 min and presented as relative fluorescence normalized to the control (n = 4). CM: conditioned media; hLEC: human lymphatic endothelial cells; KD: knockdown; kDa: kiloDaltons; shRNA: short hairpin RNA.

In order to assess the role of ezrin in Src-mediated angio/lymphangiogenesis, we utilized the human breast carcinoma MDA231 cell line expressing constitutively active pWZL-hygro-Y527F Src (MDASrc). The expression of active Src had no effect on ezrin activation (open conformation) as indicated by steady levels of phospho-Y527 ezrin (Figure 1A, left panel). Stable ezrin KD was achieved by expression of a pLKO.1 lentiviral vector containing ezrin shRNA (MDASrc shEZR) (Figure 1A, right panel). The expression of ezrin shRNA had no significant effect on the proliferation of MDASrc cells (Figure 1B). Next, we examined the role of Src/ezrin in tumour-induced migration and tube formation of primary hLEC *in vitro*. hLEC co-cultured with ezrin-deficient cells displayed significant reduction in migration compared to co-culture with MDASrc cells (Figure 1C). In the tube formation assay, CM collected from MDASrc shEZR cells reduced the ability of hLEC to form vessel-like structures in comparison to the MDASrc group (Figure 1D). The diffusion of TMR-dextran beads (2000 kDa) across a lymphatic barrier (hLEC embedded in collagen) was utilized to measure the effect of cancer cells on vascular permeability (Figure 1E). The diffusion of TMR-dextran across the lymphatic barrier co-cultured with MDASrc shEZR cells was significantly decreased when compared to MDASrc cells (Figure 1F). Examination of cell-cell junctions in hLEC, by staining for ZO-1, further corroborated these findings (Figure S1 in Additional file 1).

### Ezrin KD inhibits Src-induced angiogenic activity

**Figure 2:**
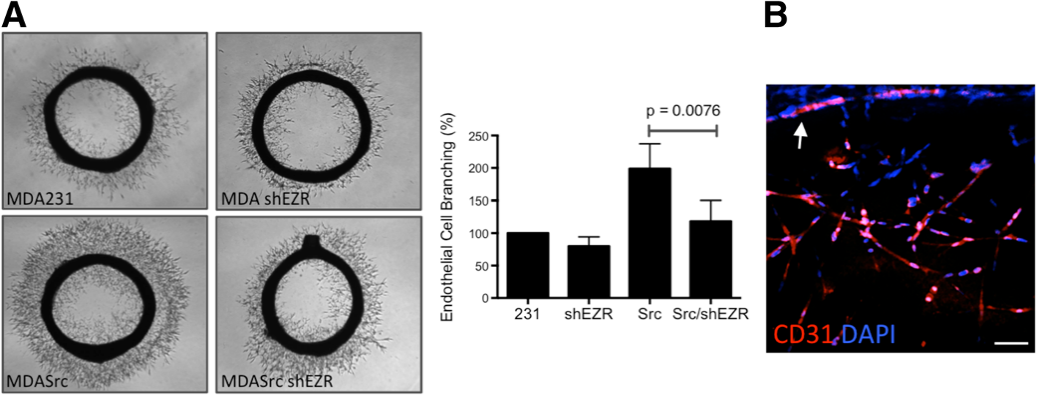
**Ezrin KD inhibits tumour-induced endothelial cell sprouting. (A)** Aortic rings were stimulated with CM from MDA231 (231), MDA shEZR (shEZR), MDASrc (Src), and MDASrc shEZR (Src/shEZR) for 96 hr prior to imaging. Endothelial cell sprouting was quantified using ImagePro software and presented as percentage of control (bar graph; n = 4 in triplicates). ANOVA post-test analysis revealed MDASrc as the only group with significantly higher angiogenic activity. **(B)** Three-dimensional reconstruction of confocal images of CD31-positive endothelial cells migrating out of aortic ring (Scale bar = 200 μm). CM: conditioned media; KD: knockdown.

Angiogenic properties of CM collected from MDASrc and MDASrc shEZR cells were analyzed by stimulating endothelial branching in Matrigel-embedded aortic ring explants. The expression of active Src induced a robust angiogenic response in aortic rings treated with CM from MDASrc compared to MDA231 cells (Figure 2A). However, ezrin KD was able to significantly reduce the angiogenic effect of MDASrc cells. Ezrin KD in MDA231 cells, expressing endogenous WT Src and therefore less angiogenic than MDASrc cells, also revealed noticeable reduction in angiogenic activity compared to the MDA231 control, although the effect did not reach statistical significance due to the short timeline of this assay. Migrating cells from the aorta rings were predominately endothelial cells as indicated by CD31 staining (Figure 2B and Movie S1 in Additional file 2).

### Ezrin is required in tumour-induced angio/lymphangiogenesis *in vivo*

**Figure 3:**
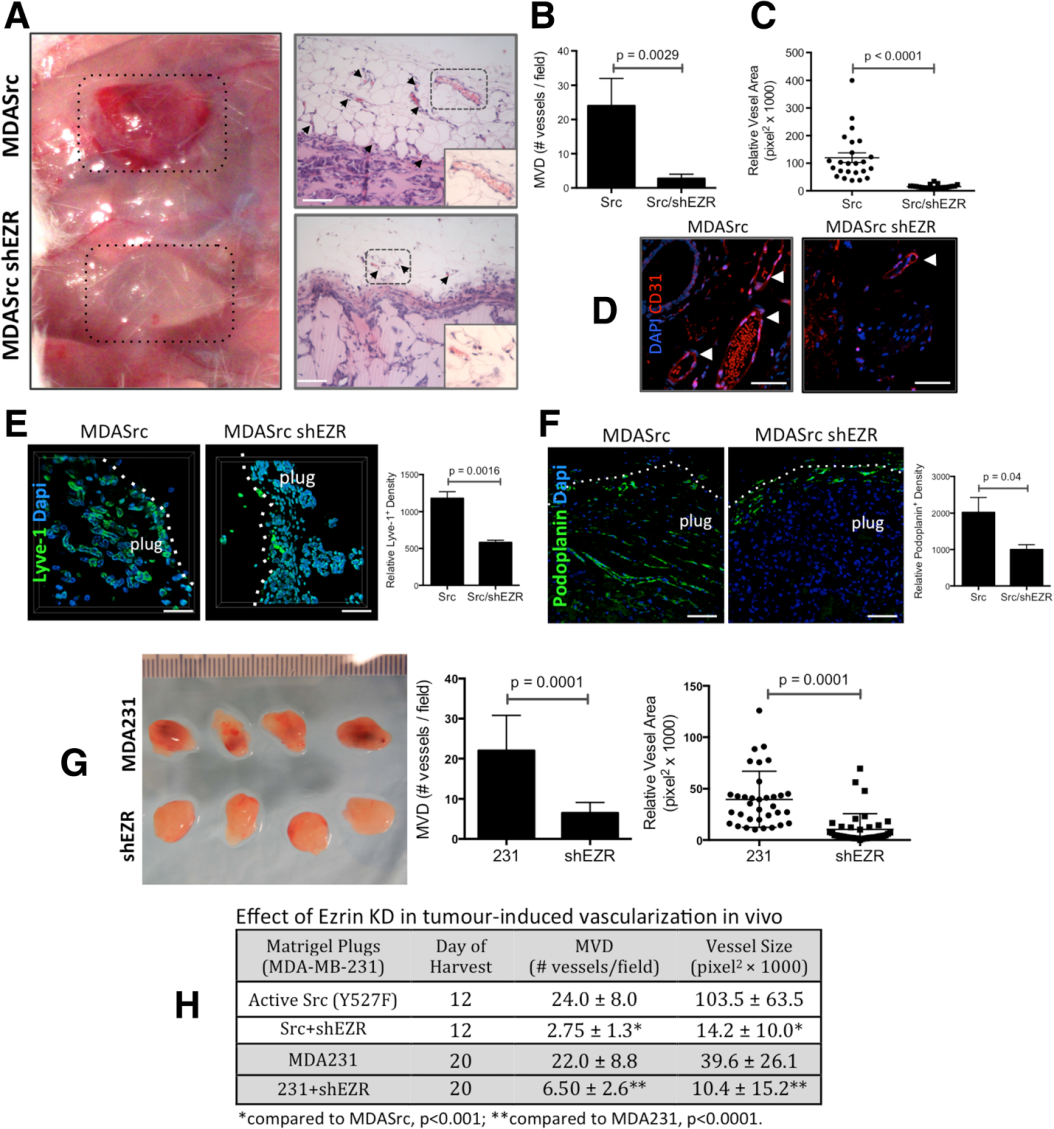
**Ezrin KD inhibits tumour-induced angio/lymphangiogenesis** ***in vivo*** **. (A)** Matrigel plugs containing MDASrc (Src) or MDASrc shEZR (Src/shEZR) were digitally photographed on day 12 post injection in Rag2γ mice. Tumour-induced angiogenesis was analyzed in Matrigel plugs H&E sections for **(B)** MVD per selected field (manual count by trained pathologist blinded to the experiment), and **(C)** vessel luminal area (dots represent the mean vascular area in each selected field in pixel^2^). **(D)** Nature of blood vasculature was confirmed by CD31 immunostaining. **(E and F)** To assess tumour-induced lymphangiogenesis, Matrigel plugs were stained for lymphatic markers Lyve-1 and podoplanin followed by quantification of relative fluorescence density of each marker (bar graphs). **(G)** The Matrigel plugs derived from MDA231 (231) and MDA shEZR (shEZR) were harvested on day 20 post injection and assessed for MVD and vessel lumen area in corresponding H&E sections. **(H)** The effect of ezrin KD on MVD and vessel lumen size in tumours derived from MDA231 and MDASrc harvested on days 12 and 20 is summarized. Arrowheads in panels A and D point to microvessels in the tumour H&E sections. Inserts in panel A represent higher magnifications (×400) of areas in dotted boxes. *P* values were obtained from unpaired *t* test statistical analysis. All scale bars = 200 μm, except panel D bar = 50 μm. H&E: hematoxylin and eosin; KD: knockdown; MVD: microvessel density; Rag2γ: Rag2^-/-^II2rg^-/-^ mice.

To compare the angio/lymphangiogenic potential of MDASrc and MDASrc shEZR cells *in vivo*, we performed a Matrigel plug xenograft assay in mice (Figure 3A). The expression of both Src and ezrin KD were confirmed by immunostaining of tumour sections (Figure S2 in Additional file 3). Tumours containing ezrin-deficient cells displayed a five-fold reduction in vascular density and a 10-fold reduction in vascular luminal area compared to MDASrc plugs (Figure 3B and C, respectively). Strong CD31 staining confirmed the presence of blood vessels in the tumour (Figure 3D). Tumours derived from ezrin-deficient cells demonstrated a significant reduction in lymphangiogenesis compared to MDASrc cells, as indicated by lymphatic vessel markers lyve-1 and podoplanin (Figure 3E and F, respectively). Furthermore, intravital microscopy revealed a robust and functional vascular network invading the MDASrc tumours, whereas vessels in the ezrin-deficient tumours were only found at the tumour periphery (Movie S2 in Additional file 4). Ezrin KD in the presence of endogenous Src expression in MDA231 cells also significantly inhibited tumour-induced vascularization (Figure 3G). The table in Figure 3H summarizes the effect of ezrin KD on tumour-induced vascularization in the presence or absence of constitutively activated Src. An independent ezrin shRNA vector expressed in MDASrc cells yielded comparable results (Figure S3, panel A in Additional file 5). In addition, repeat of the above Matrigel plug assay in nude (Figure S4 in Additional file 6) and immunocompetent (syngeneic AC2M2 graft) CBA/J mouse recipients yielded results similar to Rag2γ mice (Figure S3, panels B-D in Additional file 5).

### Y477F ezrin inhibits tumour-induced angio/lymphangiogenesis

**Figure 4:**
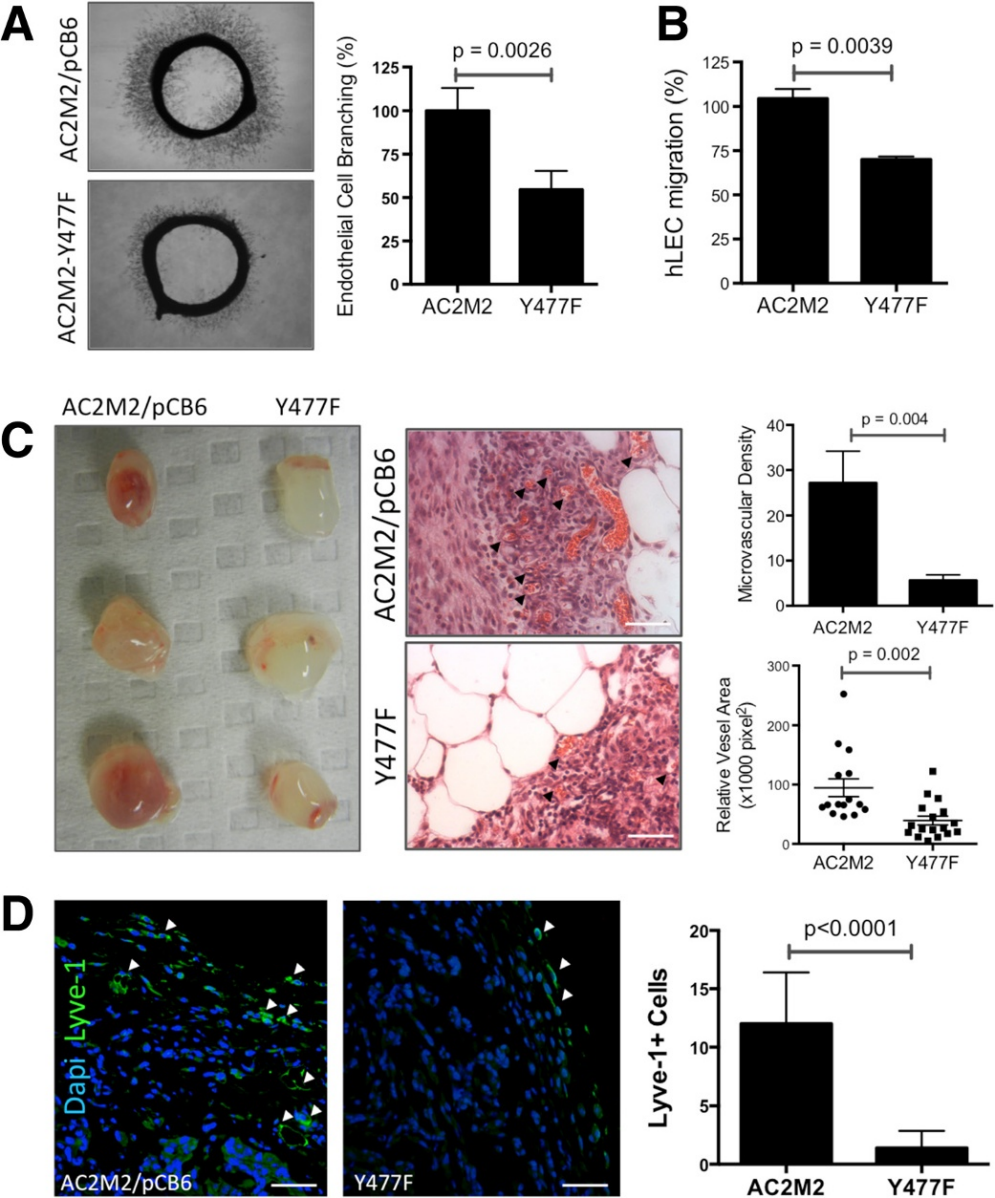
**Expression of Y477F mutant ezrin abrogates tumour-induced angio/lymphangiogenesis. (A)** Aortic rings were stimulated with CM from AC2M2 cells expressing pCB6 empty vector or Y477F ezrin and endothelial sprouting imaged (96 hr) and analyzed by ImagePro software (n = 3, bar graph). **(B)** Transwell migration of hLEC co-cultured with AC2M2 or Y477F cells (6 hr) was assessed by automatic count (ImageJ) of cells stained with DAPI. **(C)** Syngeneic Matrigel plug assay of AC2M2 or Y477F cells in CBA/J mice photographed on day 12 post injection along with representative H&E sections (arrows point to microvessels). Angiogenic activity was analyzed by MVD and vessel cross-sectional area (graphs to the right). **(D)** Lyve-1-positive cells (arrows) in plugs were quantified in selected hot spots by pathologist blinded to the study. *P* values were obtained from unpaired *t* test statistical analysis. Scale bars = 50 μm. CM: conditioned media; H&E: hematoxylin and eosin; MVD: microvessel density.

Ezrin Y477, a Src substrate, is required for the Src-induced invasive phenotype in breast cancer cells [11],[12]. Therefore, we examined the angio/lymphangiogenic potential of AC2M2 cells expressing the phosphorylation-deficient Y477F ezrin mutant, which competes with endogenous WT ezrin (EZR/WT) [12]. Aortic rings stimulated with CM from Y477F mutant-expressing cells displayed significant reduction in endothelial cell branching in comparison to AC2M2 cells expressing the pCB6 empty vector (Figure 4A). Furthermore, hLEC co-cultured with Y477F cells demonstrated reduced mobility when compared with control cells in a Transwell migration assay (Figure 4B). Engraftment of Y477F expressing AC2M2 cells into immunocompetent syngeneic CBA/J mice revealed significant reductions in tumour-induced angio/lymphangiogenesis (Figure 4C and D, respectively).

### Ezrin is required for Src-induced Stat3 phosphorylation and VEGF-A/-C and IL-6 expression

**Figure 5:**
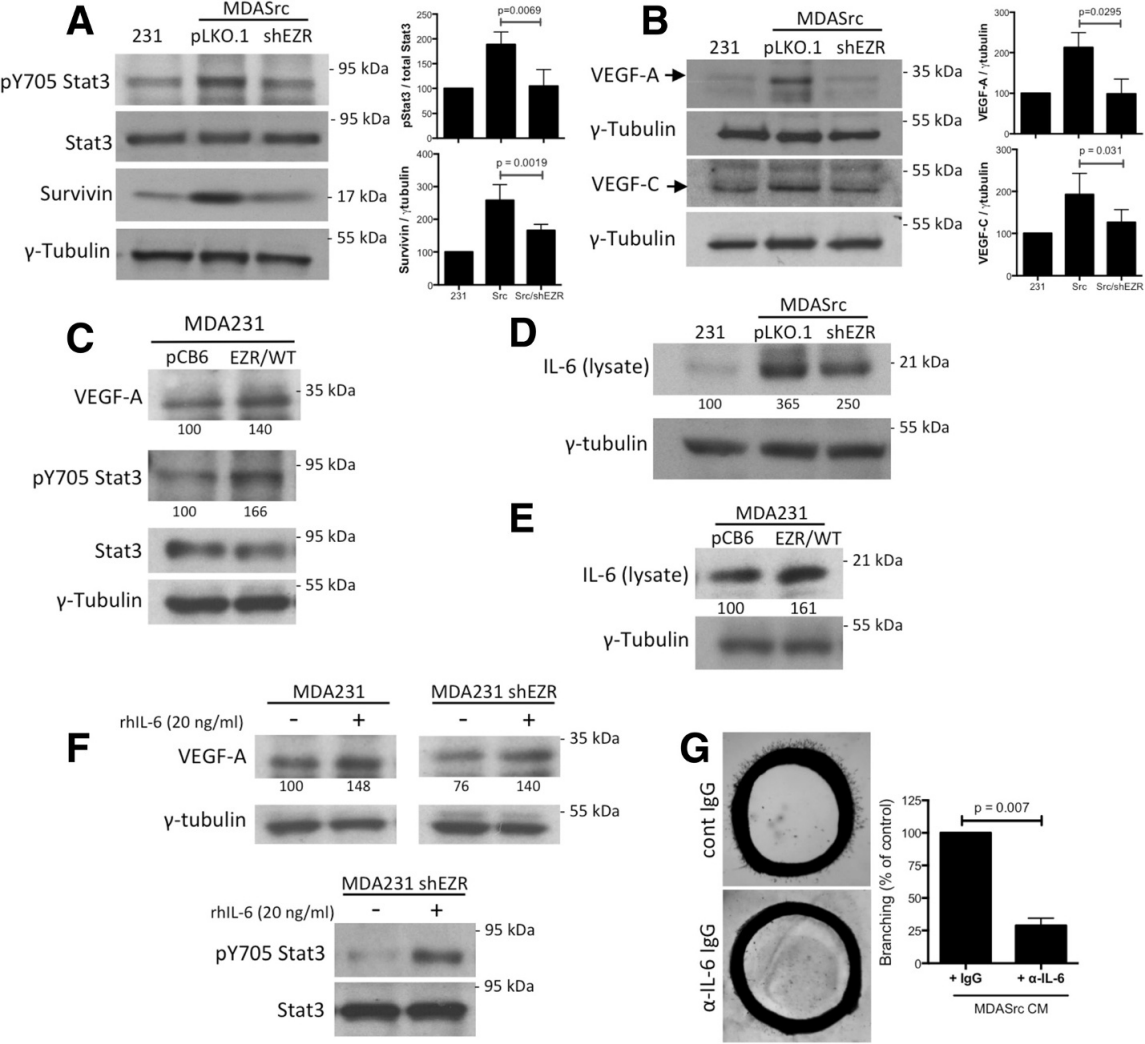
**Ezrin KD blocks Stat3 phosphorylation, VEGF-A/-C, and IL-6 expression.** Representative western blots display pY705-Stat3, Stat3, and survivin **(panel A)** and VEGF-A/-C **(panel B)** levels in MDA231, MDASrc, and MDASrc shEZR lysates. γ-tubulin expression was used as loading control. Densitometry results are presented as an average (n = 3) ratio of target protein to γ-tubulin and normalized against MDA231 controls (bar graphs). *P* values were calculated using unpaired *t* test. **(C)** Cell lysates from MDA231 cells expressing the empty vector pCB6 or wild-type ezrin (EZR/WT) were analyzed for VEGF-A, pY705 Stat3, and Stat3 levels. **(D)** Immunoblot analysis of IL-6 in cell lysates from MDA231 and MDASrc cells expressing the empty vector pLKO.1 or shEZR vector. **(E)** Immunoblot analysis of IL-6 levels in cell lysate from MDA231 cells expressing the empty vector pCB6 or wild-type ezrin (EZR/WT). **(F)** Immunoblot analysis of VEGF-A, pY705 Stat3, and Stat3 in MDA231 shEZR cells treated with vehicle alone or 20 ng/ml recombinant human IL-6 for 20 hr. **(G)** The angiogenic activity of CM collected from confluent MDASrc in the presence of α-IL-6 neutralizing antibody or non-immune IgG was assessed using the aortic ring assay. Endothelial cell sprouting was quantified using ImagePro software and presented as percentage of control (bar graph). Identical amount of α-IL-6 antibody was added to rings in the MDASrc group to control for the direct effect of antibody on endothelial cell sprouting. Panels C-F: relative densitometry values are displayed under corresponding bands (normalized against γ-tubulin). CM: conditioned media; IgG: immunoglobulin G; IL-6: interleukin-6; KD: knockdown; Stat3: signal transducer and activator of transcription 3; VEGF: vascular endothelial growth factor.

Src has been shown to induce VEGF expression through Stat3 activation [24],[25]. However, it is not known whether ezrin is required for Src regulation of the Stat3/VEGF pathway. Our results show that Src-induced increases in Stat3 phosphorylation (pY705), survivin expression (Stat3 target), and VEGF-A/-C expression were reversed in ezrin-deficient MDASrc cells (Figure 5A,B). A similar pattern was observed in CM from MDASrc and MDASrc shEZR cells (Figure S5, panel A in Additional file 7). Furthermore, overexpression of wild-type ezrin (EZR/WT) in the absence of constitutively active Src in MDA231 cells revealed an increase in pY705 Stat3 phosphorylation and VEGF expression, compared to empty vector (Figure 5C). To investigate the effect of ezrin KD on endogenous Src activity (enriched in the cytoskeletal compartment [16]), the insoluble fractions of MDA231 cell lysates were analyzed for pY416 Src levels. As shown in Figure S5, panel C in Additional file 7, depletion of ezrin by shRNA significantly decreased Src pY416 phosphorylation without an effect on Src expression.

Proteomic analysis of MDA231 CM identified IL-6 as a cytokine regulated by ezrin (data not shown) and the IL-6/Stat3 signaling loop has been reported to promote angiogenesis and metastasis in breast cancer [26]. Our results show that elevated IL-6 expression in MDASrc cells was markedly reduced following ezrin KD (Figure 5D). Ezrin depletion in MDA231 cells, expressing endogenous WT Src, also diminished IL-6 levels in cell lysate and CM (Figure S5, panel B in Additional file 7). Interestingly, the inhibitory effect of ezrin KD on VEGF and Stat3 phosphorylation can be rescued by the addition of recombinant human IL-6 (Figure 5F). Moreover, overexpression of EZR/WT increased IL-6 expression compared to MDA231 cells expressing an empty vector (Figure 5E). Next, we examined the effect of blocking IL-6 by collecting CM from MDASrc cells in the presence of an IL-6 neutralizing antibody [27]. The presence of IL-6 antibody, compared to non-immune IgG-treated CM, significantly reduced the angiogenic potential of MDASrc CM in an aortic ring assay (Figure 5G).

### Ezrin and Src expressions correlate with lymphovascular invasion in breast cancer

**Figure 6:**
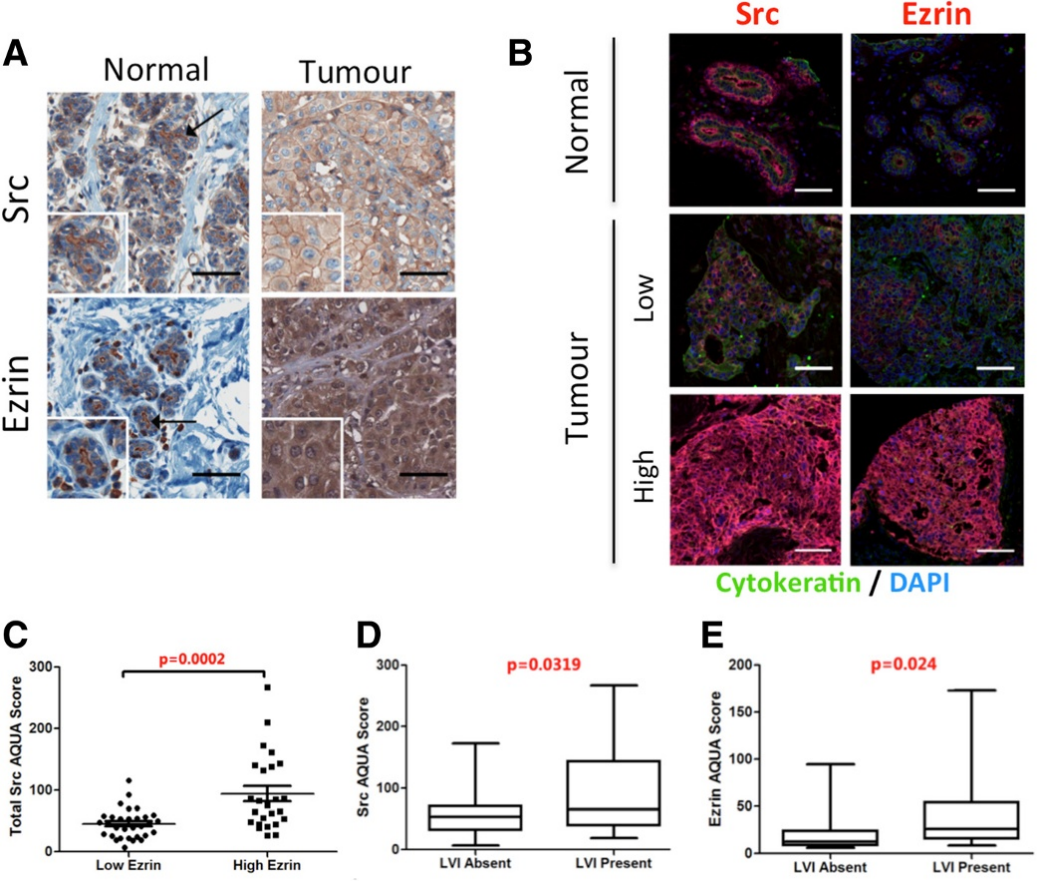
**Src and ezrin expression in human breast cancer. (A)** IHC staining of breast normal and tumour tissue was used to validate Src and ezrin antibodies. Scale bars = 75 μm. **(B)** Cy5 IF staining was used to assess the expression levels of Src and ezrin in the 63-patient breast cancer TMA. Representative tumour cores with high and low expression of Src and ezrin are presented. Images were acquired using Aperio ScanScopeFL. Scale bars = 100 μm. **(C)** Association between dichotomized ezrin AQUA scores (cutoff: median) and continuous Src AQUA scores based on IF-stained 63-TMA. **(D, E)** Continuous AQUA scores of total Src and ezrin were correlated against dichotomized LVI status. Statistical associations were assessed using the unpaired Student's *t* test. Associations were considered significant when *P* <0.05. AQUA: automated quantification analysis IF: immunofluorescence; IHC: immunohistochemistry; LVI: lymphovascular involvement; TMA: tissue microassay.

Primary breast tumour cores from a nested cohort of a 63-patient TMA were assessed for total Src and ezrin expression using an IF-based AQUA. IHC staining was initially used to validate Src and ezrin antibodies and to assess their localization in normal and tumour breast tissue. Src and ezrin exhibited a strong apical staining in breast ductal epithelium and diffuse cytoplasmic staining in the breast tumour (Figure 6A). Multiplex IF staining with cytokeratin (epithelial marker) allowed for quantification of Src and ezrin expressions in the epithelial compartment and exclusion of the stroma region. As shown in Figure 6B, a diverse range of Src and ezrin expression was detected in the tumour tissues. Dichotomized high ezrin AQUA scores (based on median) revealed a significant association between high Src and ezrin expression (Figure 6C). Furthermore, the correlation analysis between continuous AQUA scores and dichotomized clinicopathological data revealed that high expression of Src and ezrin were significantly associated with LVI in breast cancer (Figure 6D and E, respectively). A trend between high ezrin expression and positive LN status was also suggested, but did not reach statistical significance (not shown).

## Discussion

We and others have previously shown an intrinsic regulatory role for ezrin in cancer invasion and metastasis [11],[12],[28]-[30]. Despite the growing evidence pointing to ezrin as a key promoter of tumour metastasis, the molecular basis of ezrin function remains poorly understood. In the present study, we demonstrate that ezrin-deficient cells display a significant reduction in VEGF-A/-C expression and angio/lymphangiogenic activity, thus indicating a novel extrinsic mechanism by which ezrin regulates early stages of metastasis. We further show that ezrin tyrosine 477, a Src phosphorylation site, plays a regulatory role in tumour-induced angio/lymphangiogenesis. Our findings modify previously described mechanisms in Src-induced tumour angiogenesis [13] by defining a requirement for ezrin and its phosphorylation by Src in this process. We suggest that the overexpression of Src and ezrin, commonly observed in a variety of cancers, promotes metastasis by inducing tumour blood and lymphatic vascularization in addition to previously described induction of invasive phenotype in tumour cells [11],[12].

The novel role for ezrin in the regulation of tumour lymphangiogenesis is consistent with previous clinical reports suggesting a strong association between ezrin and positive LN status in breast cancer [3],[4]. Our results suggest that a positive correlation exists between high ezrin expression and LVI in breast cancer, providing a rational for the presence of LN metastasis in tumours with high ezrin expression [4],[31]. A trend of association between high ezrin expression and positive LN status was also suggested in our TMA analysis and is currently being validated in a larger breast cancer cohort. Furthermore, the majority of breast tumours expressing high levels of ezrin are also strongly positive for Src, thus providing clinical support for the previously described co-operative function of Src and ezrin in the experimental cancer models [11],[12],[15].

Our observed comparable growth curves for cells expressing active Src (MDASrc) and ezrin shRNA (MDASrc shEZR) exclude the possibility that the reduction in angio/lymphangiogenesis is a result of reduced proliferation rate of ezrin-deficient cells. This observation is consistent with our previous demonstration that expression of mutant ezrin Y477F does not inhibit growth in three-dimensional cell colonies or primary tumour xenografts [12]. Moreover, we observed no significant difference in the growth rate of MDASrc and MDASrc shEZR tumours in a Rag2γ xenograft mammary fat pad model (unpublished data). Osawa *et al*. also demonstrated, using a medulloblastoma model, that the overexpression of ezrin promotes invasion without causing a change in the proliferation rate of cancer cells [32].

Tumour-associated immune cells play a critical role in the promotion of neovascularization [33]. Therefore, in addition to the Rag2γ model (T, B, NK cell deficient), we examined the effects of ezrin KD and Y477F mutation in nude (mature T cell deficient) and immunocompetent histocompatible CBA/J mouse models. All three animal models yielded similar reductions in vascularization of ezrin-deficient or Y477F ezrin-expressing tumour cells. Together, these findings support the requirement of ezrin in tumour-induced angio/lymphangiogenesis regardless of T, B, and NK cell status of host recipients.

An indirect link between Src activity and lymphangiogenesis was previously implied by an observed reduction in VEGF-C secretion, a potent lymphangiogenic factor [34], in MDA231 cells treated with the Src kinase inhibitor, PP1 [35]. Our results identify a direct requirement for ezrin in Src-induced VEGF-C expression in breast carcinoma cells. Furthermore, our demonstrated regulatory role of ezrin in the expression of IL-6 is highly relevant as high levels of IL-6 correlate with increased metastatic potential and poor outcome in breast cancer [36]. A signaling pathway linking IL-6 with Stat3 activation and VEGF expression has been previously described [37]. In addition, Chang *et al*. have recently described a regulatory role for a Stat3/IL-6 positive feed-forward loop in promoting angiogenesis and metastasis [26]. Our findings further build on these models by suggesting a role for ezrin in the regulation of IL-6 expression and Stat3 activation leading to the upregulation of VEGF-A/-C and tumour angio/lymphangiogenesis. As rhIL-6 was able to induce Stat3 activation and VEGF-A expression in ezrin-deficient cells, we believe that ezrin operates upstream of IL-6/Stat3 by positively regulating IL-6 transcription. Interestingly, nuclear factor κB activity, an important transcription factor in activation of IL-6 gene, requires ezrin binding in the regulation of IκB phosphorylation [30].

The exact mechanism by which ezrin and Src interact to regulate the Stat3/VEGF pathway is not fully understood. Src binds via its SH2 domain to pY190 ezrin and phosphorylates ezrin at Y145, resulting in a reciprocal stabilization of Src activity [14]. A similar regulation of Src activity has been reported for other Src substrates, such as focal adhesion kinase (FAK) [38], the tyrosine phosphatase Shp-2 [39], and the adapter protein p130^CAS^ (CAS) [40]. Src also phosphorylates ezrin Y477 [9], a tyrosine residue unique to this ERM family member. Heiska *et al*. and our group have recently demonstrated a role for ezrin Y477 in Src-induced anchorage-independent invasive growth in three-dimensional environment [11] and in local invasion and metastasis in a breast cancer xenograft model [12]. This result is in line with our observed reduction in angio/lymphangiogenesis in tumours derived from ezrin Y477F-expressing cells. The reduction of Src activity (pY416) in ezrin-deficient cells following short-term (2 hr) seeding on a collagen-coated surface, suggests a requirement for ezrin in Src activation. Considering ezrin's role as a crosslinker and scaffold protein, it is plausible for ezrin to act as a docking site for Src and its regulators.

Hepatocyte growth factor (HGF) and its receptor Met play a significant role in tumour progression partly through upregulation of Src, Stat3 activity, and VEGF expression [41],[42]. HGF/Met-mediated Stat3 activation can in turn upregulate the expression of IL-6 [43], which feeds into the Stat3/IL-6 forward loop [26]. Interestingly, Y477 ezrin phosphorylation is required by Src in HGF-induced scattering of epithelial cells [10]. The potential involvement of HGF/Met in Src/ezrin-mediated tumour angio/lymphangiogenesis is consistent with our recent finding demonstrating Src/ezrin co-operation increased Met activation and extracellular matrix degradation, characteristic of an invasive phenotype in breast cancer [44]. Furthermore, Zaarour *et al.* have reported a novel mechanism by which Y477 ezrin regulates the stability and activity of Met receptor, by interacting with the ubiquitin ligase WWP1, which is overexpressed in human breast and prostate cancers [45]. Therefore, ezrin silencing or Y477F mutation can result in the downregulation of Src/Met-dependent Stat3 activation followed by a reduction in IL-6/VEGF-A/-C expression and angio/lymphangiogenic activity.

## Conclusions

Further understanding of mechanisms involved in the early stages of metastatic progression is a prerequisite for discovery of novel prognostic/predictive biomarkers. The present study provides novel insights into the regulatory role of ezrin and Src in tumour-induced angio/lymphangiogenesis, a precondition for early tumour cell dissemination. Further studies are required to elucidate the role of the Src/ezrin pathway in the invasion process and its potential as a novel prognostic/predictive biomarker for early-stage metastatic phenotype in breast cancer.

## Additional files

## Electronic supplementary material

Additional file 2: **Movie S1.** Endothelial cells sprouting and migration from the aortic ring wall*.* Three-dimensional compilation of z-stacks obtained by spinning disk confocal microscope following immunostaining with endothelial cell marker CD31 (red) and DAPI (blue). Scale bar = 200 μm. (MOV 3 MB)

Additional file 4: **Movie S2.** Intravital imaging of vascular network within Matrigel plugs*.* Functional blood vessels within the Matrigel plugs were visualized by intravenous injection of FITC-albumin in anesthetized mice. Panel on the right shows time-lapse and z-stacks compilation intravital microscopy of vessels invading the MDASrc plugs. A confocal image of a vessel in the periphery of MDASrc shEZR plug is shown on the right. The dotted line represents the boundary of the plug. See Methods for further information. Scale bars = 200 μm. (MOV 4 MB)

Additional file 1: Figure S1.: Cell-cell junction marker, ZO-1, staining of hLEC barrier*.* hLEC co-cultured overnight with MDASrc or MDASrc shEZR cells were stained for the cell junction marker ZO-1 and DAPI and imaged by spinning disk confocal microscopy. In absence of ezrin expression, disruptions of hLEC tight junction, which effect vascular permeability, induced by tumour cells are markedly reduced. Scale bars = 50 μm. (PDF 561 KB)

Additional file 3: Figure S2.: Src/ezrin expression in Matrigel plugs. Matrigel plugs sections were immunostained for Src and ezrin to confirm their expression on day 12 post injection. Scale bars = 200 μm. (PDF 7 MB)

Additional file 5: Figure S3.: Validation of Ezrin shRNA knockdown. **(A)** A second ezrin shRNA (shEZR-2 in pLKO.1 vector) was used to generate a stable ezrin KD line (MDASrc shEZR-2). Repeat of Matrigel plug assay with MDASrc and MDASrc shEZR-2 cells displayed comparable reductions in tumour-induced angiogenesis as the MDASrc shEZR-1 cell line, ruling out non-specific effects of shRNA-1 ezrin KD. *P* value was obtained from unpaired *t* test analysis. **(B)** Syngeneic engraftment of Matrigel + AC2M2 cells transfected with non-silencing (siCont) or ezrin (siEZR) siRNA in CBA/J mice demonstrated significant reduction in angio/lymphangiogenesis in the presence of a fully functional immune system, as shown by MVD and vessel size assessment in H&E sections (graphs). Scale bars = 500 μm. **(C)** Lymphangiogenic activity in the same plugs (panel B) was assessed by quantification of Lyve-1-positive endothelial cells (arrows) in selected hot spots. (n = 4, at least three 'hot spots' examined per plug). Scale bars = 50 μm. **(D)** Confirmation of ezrin KD in AC2M2 by pooled siRNAs at 24, 48, and 72 hr post-transfection. A universal non-silencing siRNA (siCont) was used as control. (PDF 10 MB)

Additional file 6: Figure S4.: Matrigel plug assay in nude mice. GFR-Matrigel containing MDASrc or MDASrc shEZR cell lines were injected s.c. and harvested on day 12 post injections. Tumour-induced angiogenic activity was assessed by MVD and vessel cross-sectional area in selected hot spots of H&E sections (n = 4). Scale bars = 200 μm. Inserts were imaged using a 40X objective. (PDF 2 MB)

Additional file 7: Figure S5.: Reduced Src activity in ezrin-deficient cells and decrease in VEGF-C/IL-6 secretion. **(A)** Representative western blot displays VEGF-C levels in conditioned media collected (24 hr) from confluent MDA231, MDASrc (pLKO.1), and MDASrc shEZR (shEZR). Densitometry analysis represents average of three blots normalized against MDA231. Our VEGF-A antibody, unlike cell lysates, did not pick up any bands in the CM, perhaps due to post-translational modification of the target epitope. **(B)** Immunoblots of IL-6 in CM and cell lysates from MDA231 (pLKO.1) and MDA231 shEZR cells. Results from densitometry analysis are shown below each band and normalized against γ-tubulin. **(C)** Following serum starvation (2 hr), suspension of cells (30 min), and seeding on collagen-I-coated plates (2 hr), soluble and insoluble fractions of MDA231 and MDA231 shEZR cell lysates were analyzed for pY416 Src, Src, pT567 ezrin (pTERM antibody), and ezrin. Src pY416 levels in insoluble fraction of MDA231 (231) and MDA231 shEZR (shEZR) cell lysates were compared by densitometry (bar graph). *P* values are calculated by unpaired *t* test. (PDF 1017 KB)

Below are the links to the authors’ original submitted files for images.

Authors’ original file for figure 1

Authors’ original file for figure 2

Authors’ original file for figure 3

Authors’ original file for figure 4

Authors’ original file for figure 5

Authors’ original file for figure 6

## Abbreviations

AQUA: automated quantification analysis
BSA: bovine serum albumin
CM: conditioned medium
DMEM: Dulbecco's modified Eagle's medium
ERM: ezrin-radixin-moesin
FBS: fetal bovine serum
GFR: growth factor-reduced
H&E: hematoxylin and eosin
HGF: hepatocyte growth factor
hLEC: human lymphatic endothelial cells
IF: immunofluorescence
IHC: immunohistochemistry
IL-6: interleukin-6
KD: knockdown
kDa: kiloDaltons
LN: lymph node
LVI: lymphovascular involvement
MTT: 3-(4,5-dimethylthiazol-2-yl)-2,5-diphenyl tetrazolium bromide)
MVD: microvessel density
Rag2γ: Rag2^-/-^II2rg^-/-^ mice
shRNA: short hairpin RNA
siRNA: small interfering RNA
Stat3: signal transducer and activator of transcription 3
TMA: tissue microarray
VEGF: vascular endothelial growth factor
WT: wild-type
ZO-1: Zonula occludens-1
