# Erratum to: “A novel role for ezrin in breast cancer angio/lymphangiogenesis”

**DOI:** 10.1186/s13058-014-0511-x

**Published:** 2015-01-23

**Authors:** Abdi Ghaffari, Victoria Hoskin, Alvin Szeto, Maaike Hum, Navid Liaghati, Kanji Nakatsu, David LeBrun, Yolanda Madarnas, Sandip SenGupta, Bruce E Elliott

**Affiliations:** Department of Pathology and Molecular Medicine, Queen’s University, Kingston, Canada; Division of Pharmacology & Toxicology, Department of Biomedical and Molecular Sciences, Queen’s University, Kingston, Canada; Department of Laboratory Medicine, The Credit Valley Hospital & Trillium Health Centre, Mississauga, Canada; Department of Oncology, Queen’s University, Kingston, Canada; Division of Cancer Biology and Genetics, Cancer Research Institute, Queen’s University, Botterell Hall, 18 Stuart St, Kingston, ON K7L 3 N6 Canada

## Erratum

Following the publication of our article [1], we noticed that Dr. David LeBrun had inadvertently been omitted from the author list. DL declares he has no conflicts of interest. The author list has now been corrected and the amended authors’ contributions included below.

AG performed the majority of experiments, analyzed the data, and drafted the manuscript. VH provided MDA231 and MDASrc ezrin KD and EZR/WT-expressing cell lines and contributed to the design of experiments. AS performed the TMA staining for Src/Ezrin, image analysis, clinical outcome correlations, and VEGF-A/-C western blots. DL supervised AS in establishing and optimizing the quantification of Src/ezrin in histopathological tissue samples by multi-channel immunofluorescence. MH assisted with the aortic rings experiment and analysis. NL assisted in quantification of angio/lymphangiogenic activity in tumour xenograft H&E sections. KN assisted in design and interpretation of aortic ring assay and analysis. YM collected the clinical outcome data for the in-house human breast cancer cohort and assisted in analysis and interpretation of clinical correlative studies. SS assessed the tumours in our breast cancer cohort and oversaw the construction, staining, and analysis of all TMA-related experiments. BEE conceived the project, supervised experiments & analysis, and contributed to writing of the final manuscript.

All authors have made substantial contribution to the design of experiments, data analysis, and drafting of the manuscript. All authors have reviewed and approved the final version of this manuscript and agree to be accountable for all aspects of the work presented herein.
