# Supplementary figures and images for: A novel role for ezrin in breast cancer angio/lymphangiogenesis
Source: Breast Cancer Res. 2014 Sep 18;16:438. doi: 10.1186/s13058-014-0438-2 (PMC4303119; doi:10.1186/s13058-014-0438-2)

Figure S1-Cell-cell junction marker ZO-1 staining  
of lymphatic barrier

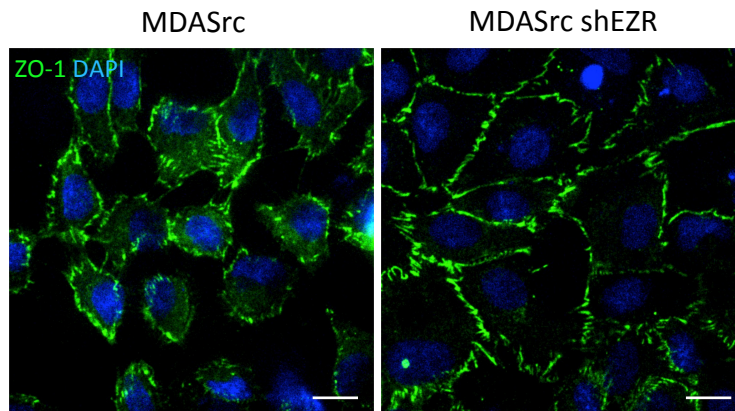

Supplement: Supplementary file 3 — Additional file 1: Figure S1.: Cell-cell junction marker, ZO-1, staining of hLEC barrier. hLEC co-cultured overnight with MDASrc or MDASrc shEZR cells were stained for the cell junction marker ZO-1 and DAPI and imaged by spinning disk confocal microscopy. In absence of ezrin expression, disruptions of hLEC tight junction, which effect vascular permeability, induced by tumour cells are markedly reduced. Scale bars = 50 μm. (PDF 561 KB) [file 13058_2014_438_MOESM1_ESM.pdf]

Figure S2-Src and ezrin expression in *Matrigel plugs*

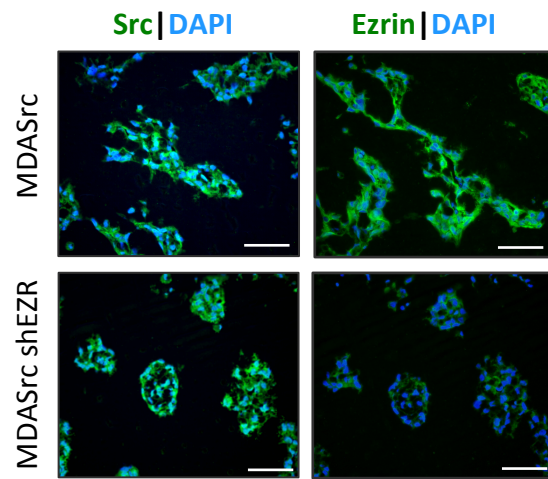

Supplement: Supplementary file 4 — Additional file 3: Figure S2.: Src/ezrin expression in Matrigel plugs. Matrigel plugs sections were immunostained for Src and ezrin to confirm their expression on day 12 post injection. Scale bars = 200 μm. (PDF 7 MB) [file 13058_2014_438_MOESM3_ESM.pdf]

# Figure S3-shRNA and siRNA knockdown of ezrin in Matrigel plug assay

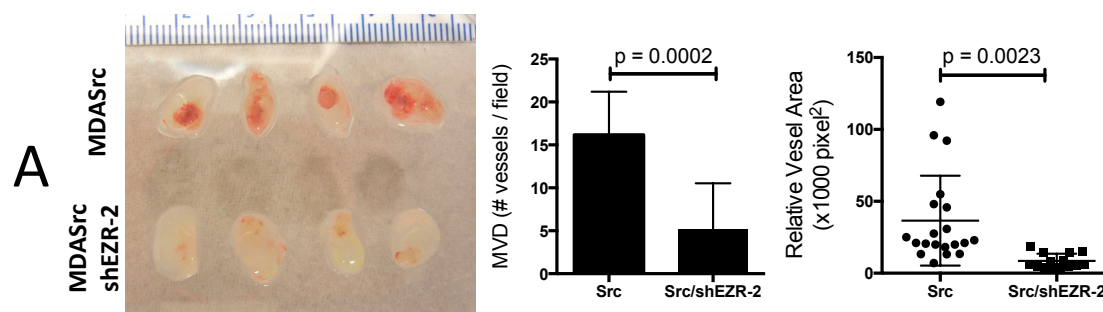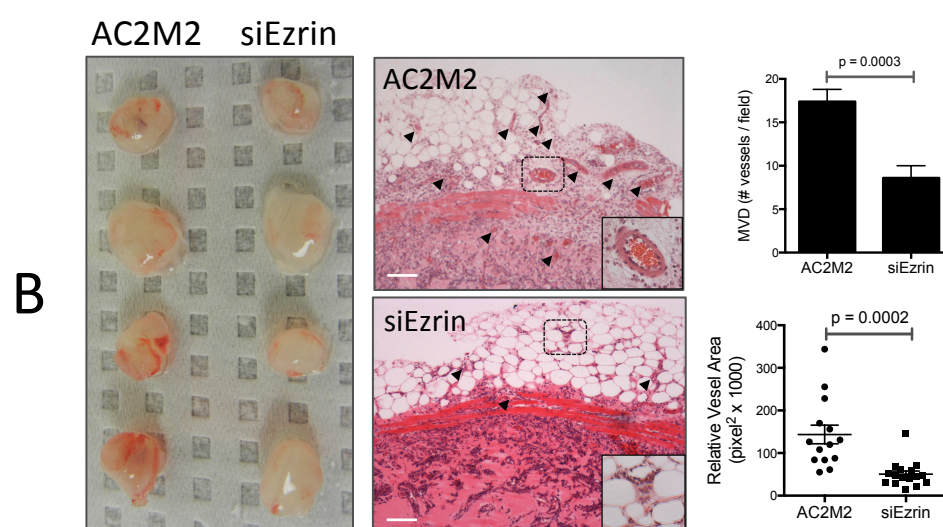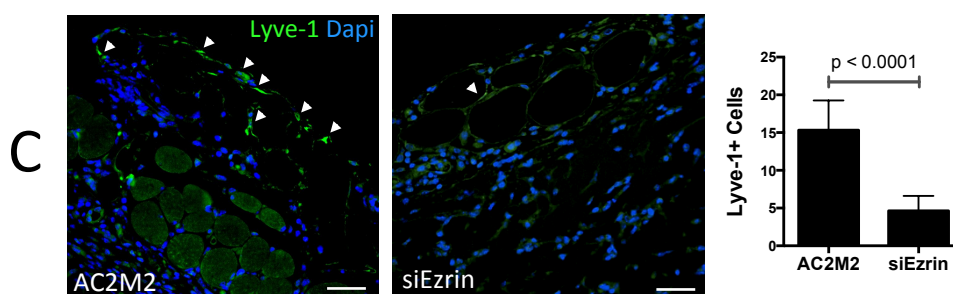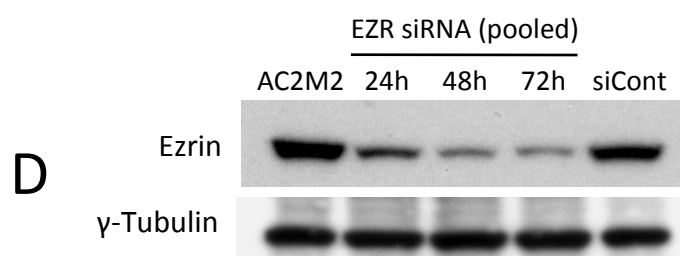

Supplement: Supplementary file 5 — Additional file 5: Figure S3.: Validation of Ezrin shRNA knockdown. (A) A second ezrin shRNA (shEZR-2 in pLKO.1 vector) was used to generate a stable ezrin KD line (MDASrc shEZR-2). Repeat of Matrigel plug assay with MDASrc and MDASrc shEZR-2 cells displayed comparable reductions in tumour-induced angiogenesis as the MDASrc shEZR-1 cell line, ruling out non-specific effects of shRNA-1 ezrin KD. P value was obtained from unpaired t test analysis. (B) Syngeneic engraftment of Matrigel + AC2M2 cells transfected with non-silencing (siCont) or ezrin (siEZR) siRNA in CBA/J mice demonstrated significant reduction in angio/lymphangiogenesis in the presence of a fully functional immune system, as shown by MVD and vessel size assessment in H&E sections (graphs). Scale bars = 500 μm. (C) Lymphangiogenic activity in the same plugs (panel B) was assessed by quantification of Lyve-1-positive endothelial cells (arrows) in selected hot spots. (n = 4, at least three 'hot spots' examined per plug). Scale bars = 50 μm. (D) Confirmation of ezrin KD in AC2M2 by pooled siRNAs at 24, 48, and 72 hr post-transfection. A universal non-silencing siRNA (siCont) was used as control. (PDF 10 MB) [file 13058_2014_438_MOESM5_ESM.pdf]

Figure S4-*Matrigel plus assay in Nude mice*

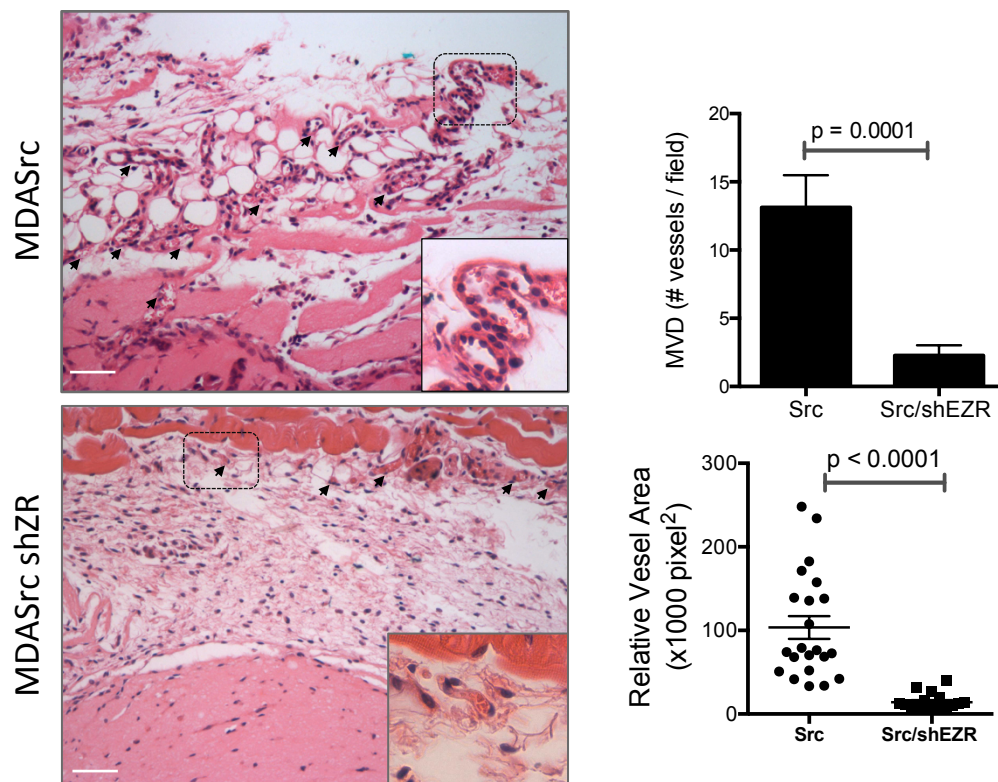

Supplement: Supplementary file 6 — Additional file 6: Figure S4.: Matrigel plug assay in nude mice. GFR-Matrigel containing MDASrc or MDASrc shEZR cell lines were injected s.c. and harvested on day 12 post injections. Tumour-induced angiogenic activity was assessed by MVD and vessel cross-sectional area in selected hot spots of H&E sections (n = 4). Scale bars = 200 μm. Inserts were imaged using a 40X objective. (PDF 2 MB) [file 13058_2014_438_MOESM6_ESM.pdf]

# Figure S5-Reduced Src activity in *Ezrin-deficient cells* and decrease in VEGF-C / IL-6 secretion

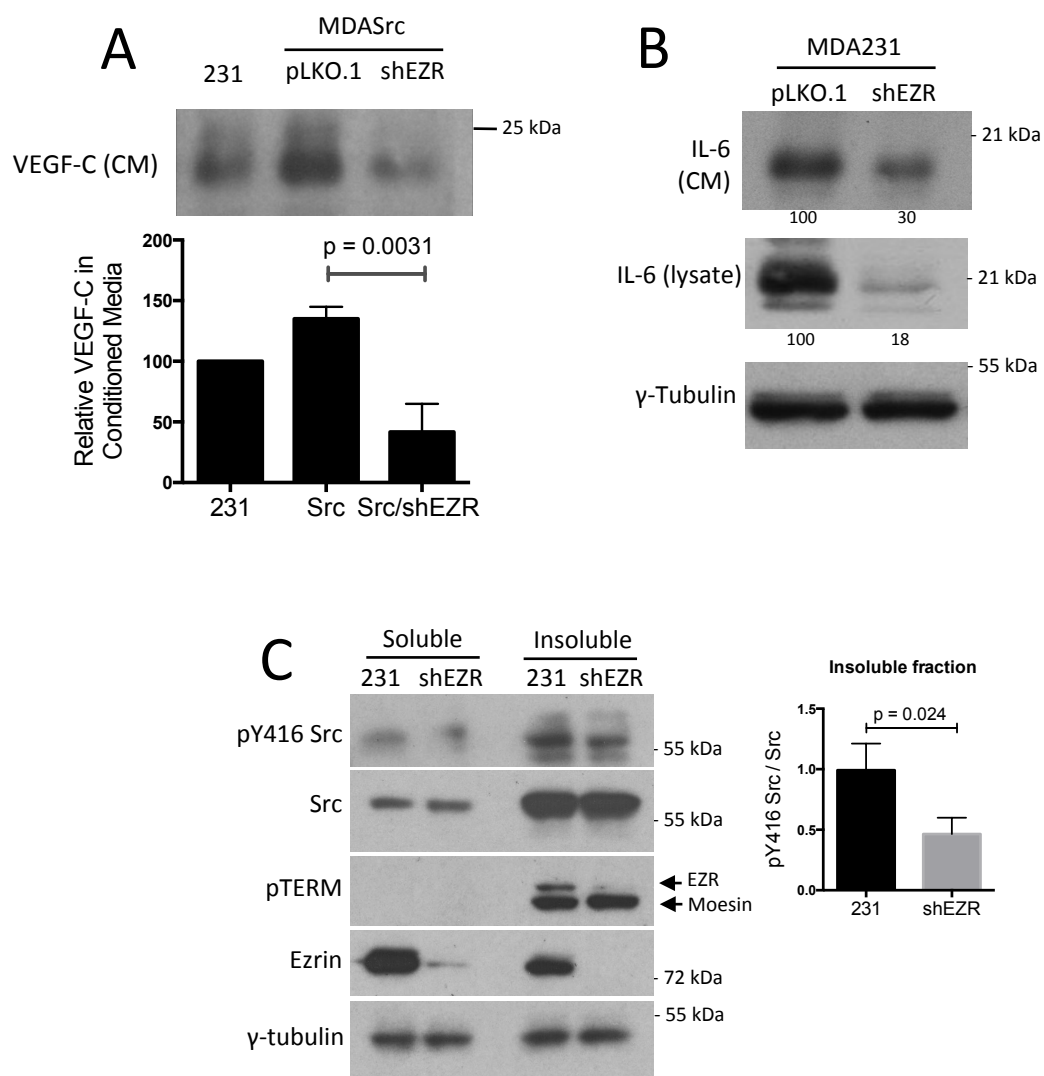

Supplement: Supplementary file 7 — Additional file 7: Figure S5.: Reduced Src activity in ezrin-deficient cells and decrease in VEGF-C/IL-6 secretion. (A) Representative western blot displays VEGF-C levels in conditioned media collected (24 hr) from confluent MDA231, MDASrc (pLKO.1), and MDASrc shEZR (shEZR). Densitometry analysis represents average of three blots normalized against MDA231. Our VEGF-A antibody, unlike cell lysates, did not pick up any bands in the CM, perhaps due to post-translational modification of the target epitope. (B) Immunoblots of IL-6 in CM and cell lysates from MDA231 (pLKO.1) and MDA231 shEZR cells. Results from densitometry analysis are shown below each band and normalized against γ-tubulin. (C) Following serum starvation (2 hr), suspension of cells (30 min), and seeding on collagen-I-coated plates (2 hr), soluble and insoluble fractions of MDA231 and MDA231 shEZR cell lysates were analyzed for pY416 Src, Src, pT567 ezrin (pTERM antibody), and ezrin. Src pY416 levels in insoluble fraction of MDA231 (231) and MDA231 shEZR (shEZR) cell lysates were compared by densitometry (bar graph). P values are calculated by unpaired t test. (PDF 1017 KB) [file 13058_2014_438_MOESM7_ESM.pdf]

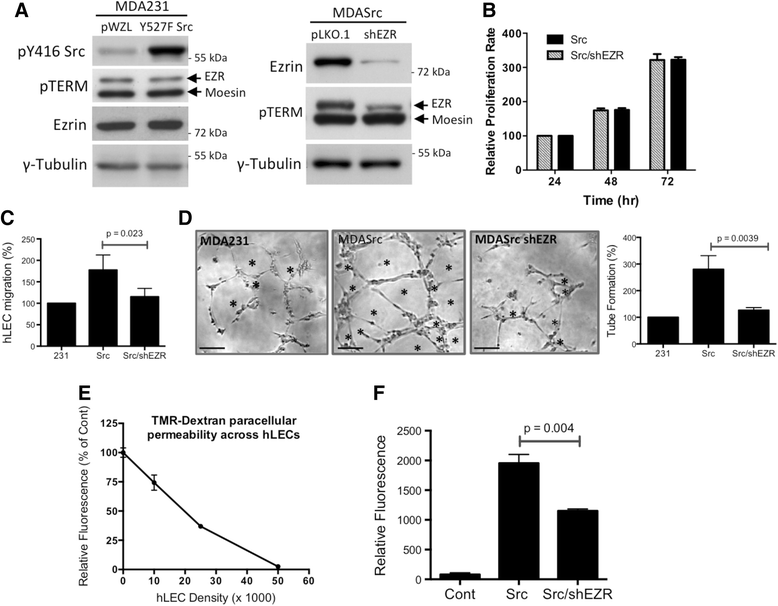

Supplement: Supplementary file 8 — Authors’ original file for figure 1 [file 13058_2014_438_MOESM8_ESM.gif]

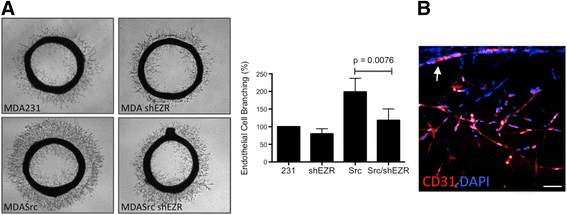

Supplement: Supplementary file 9 — Authors’ original file for figure 2 [file 13058_2014_438_MOESM9_ESM.gif]

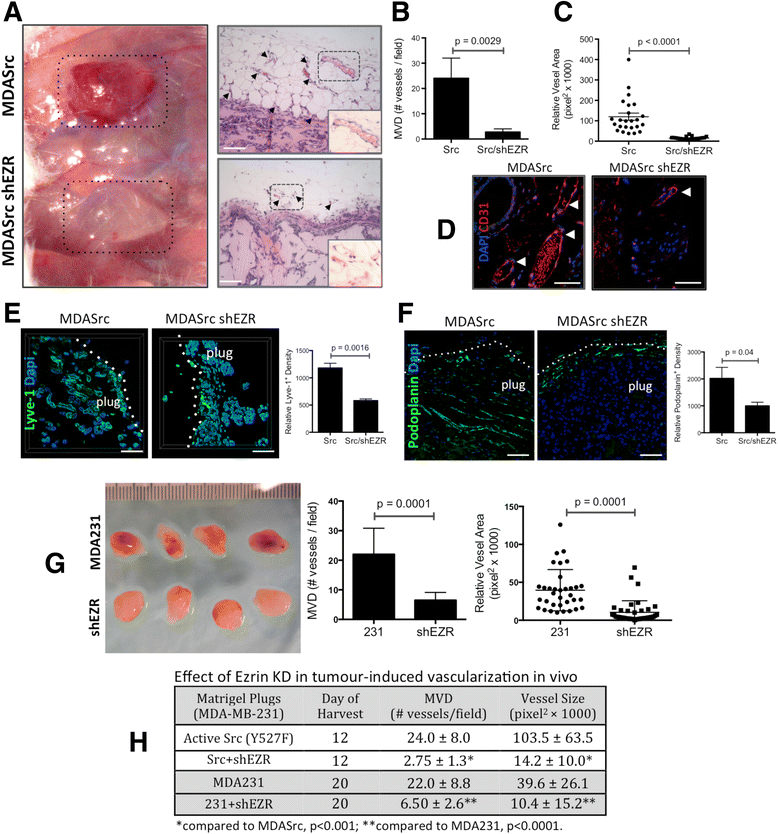

Supplement: Supplementary file 10 — Authors’ original file for figure 3 [file 13058_2014_438_MOESM10_ESM.gif]

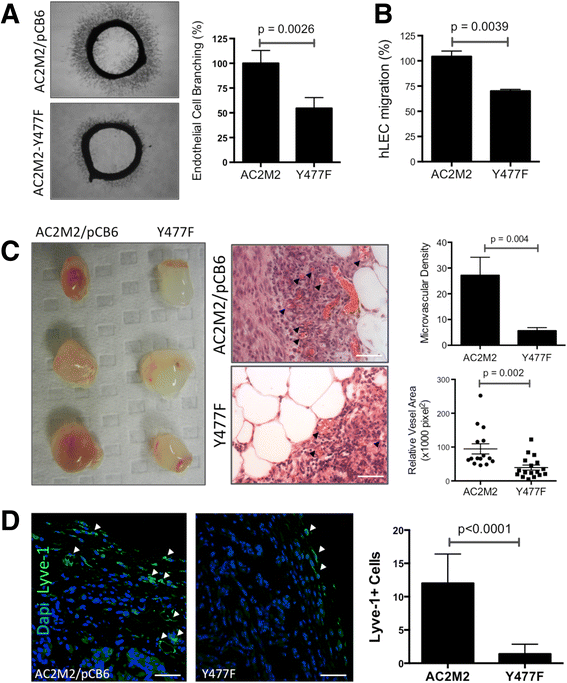

Supplement: Supplementary file 11 — Authors’ original file for figure 4 [file 13058_2014_438_MOESM11_ESM.gif]

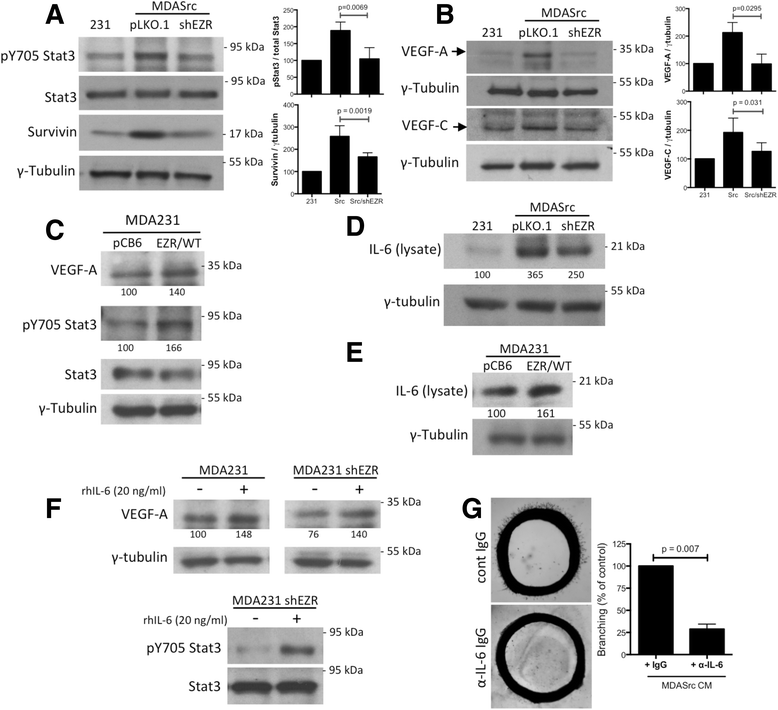

Supplement: Supplementary file 12 — Authors’ original file for figure 5 [file 13058_2014_438_MOESM12_ESM.gif]

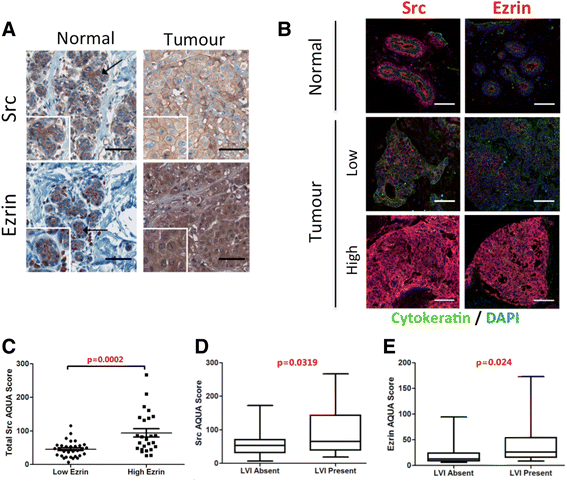

Supplement: Supplementary file 13 — Authors’ original file for figure 6 [file 13058_2014_438_MOESM13_ESM.gif]
